# Effects of Dietary Diversity on Growth Outcomes of Children Aged 6 to 23 Months in India: Evidence from National Family and Health Survey

**DOI:** 10.3390/nu15010159

**Published:** 2022-12-29

**Authors:** Jay Saha, Pradip Chouhan, Najma Iqbal Malik, Tanmoy Ghosh, Puja Das, Muhammad Shahid, Farooq Ahmed, Kun Tang

**Affiliations:** 1 Department of Geography, University of Gour Banga (UGB), Malda 732103, West Bengal, India; 2 Department of Psychology, University of Sargodha, Sargodha 40100, Pakistan; 3 Vanke School of Public Health, Tsinghua University, Beijing 100029, China; 4 School of Insurance and Economics, University of International Business and Economics (UIBE), Beijing 100029, China; 5 Department of Anthropology, Quaid-i-Azam University Islamabad, Islamabad 44000, Pakistan

**Keywords:** dietary diversity, minimum dietary diversity, children, NFHS, India

## Abstract

Low dietary diversity significantly interplays with children’s growth failure. However, evidence of its crucial role in children’s health remains inconclusive in developing countries such as India. This study attempts to find the association between dietary diversity and growth outcomes among children aged between 6 and 23 months in India using the fourth round of the National Family Health Survey (NFHS), 2015–2016. A total of 67,278 mother-child pairs of children between the ages of 6–23 months and mothers aged 15–49 years were included in this study. Pearson’s chi-square significance test and multivariable logistic regression were used to determine the association between dietary diversity and child growth outcomes (stunted, wasted, and underweight). The study found that the prevalence of stunting and severe stunting among children aged between 6 and 23 months were 35.9% and 16.2%; 23.8% and 8.5% represented wasting, and severe wasting; and more than 32%, 10% were underweight and severely underweight respectively. This present study found that having an inadequate minimum dietary diversity (<4 food groups) significantly increases the risk of being stunted (adjusted odds ratio (AOR) = 1.29; 95% confidence interval (CI); 1.21–1.38), wasted (AOR = 1.29; 95% CI; 1.21–1.38), and underweight (AOR = 1.47; 95% CI; 1.39–1.56). Further, it was noted that children who did not intake dairy products, eggs, and other fruits and vegetables were more likely to be stunted, wasted, and underweight and more likely to be severely stunted, wasted, and underweight. Therefore, additional nutrition-specific interventions are urgently needed to strengthen and enhance existing feeding interventions aimed at improving infant and young child feeding (IYCF) practices, including complementary feeding practices among children aged between 6 and 23 months in India. The Government should focus such interventions more on states or regions where the prevalence of adequate minimum dietary diversity (MDD) and malnutrition is high.

## 1. Introduction

Child undernutrition remains a significant public health challenge in the world. According to the World Health Report 2020, there are 45 million wasted children and 149 million stunted children under the age of 5 [1]. Globally, the burden of child malnutrition was substantially higher in sub-Saharan Africa (SSA) and South Asia [2]. Additionally, 50 million young children in low- and middle-income countries (LMICs) are thought to be at risk of not reaching their full developmental potential [3]. A recent data analysis discovered that about 22% of the world’s under-five children remain stunted, with most (54%) in South Asia [4].

An unequal proportion of the world’s undernourished children live in India [5,6]. India has one of the highest malnutrition burdens in south Asia, with over 3.3 million malnourished children, half of whom are classified as having severe undernutrition. The National Family Health Survey (NFHS)-5, 2019–2021, reported that 35.5% of the children were stunted, 19.3% were wasted, and 32.1% were underweight [7]. The fourth round of the NFHS conducted in 2015–2016 revealed that the majority of mothers in India are not following the recommended level of Infant and Young Child Feeding (IYCF) practices [8], also including the timely reduction of solid foods [9,10]. This might result in a stagnant rate of reduction in the prevalence of stunting, which decreased from 52% in 1993 to 38% in 2016 [9,11]. In this context, insufficient child-feeding practices are the root cause of malnutrition in India [12] and elsewhere [13]. Indeed poor nutritional status, unless dealt with properly, poses dire consequences on child health [14] since it not only causes growth faulting but also elevates the life risk of children in India [15], Ethiopia [16], and Pakistan [17].

The duration of complementary feeding, usually between 6 and 23 months, is characterized by a gradual change from breast milk to family foods [18]. It is the most vulnerable period of faltering in child growth, micronutrient deficiency, and the emergence of common childhood diseases [19]. Complementary foods often contain inadequate amounts and nutrient concentrations, which are prepared in an unhealthy way and are started too early or too late [20]. In the developing world, one-fifth of infant deaths can be avoided through optimal breastfeeding with infants who are nourished, healthy, and of the appropriate age [18]. It has been estimated that a 17% reduction in the incidents of stunting at 24 months and 6% of childhood death under the age of five can be prevented by assuring the appropriate complementary feeding practices each year [21].

Dietary diversity possesses a crucial interplay with the health status of children [22] since it is a significant protective determinant of poor nutritional status [23], which manifests itself through stunting- chronic and long-term undernutrition; wasting—an acute form of undernutrition, and underweight- a blending of both stunting and wasting among children [24]. In this regard, a diversified diet is crucial immediately after the sixth month, as exclusive breastfeeding alone can no longer meet the nutritional needs of a growing child [25].

However, as per World Health Organization’s (WHO) recommendations, daily intake of at least four food groups out of seven, namely grains, roots, and tubers; legumes and nuts; dairy products; flesh foods; eggs; vitamin A-rich fruits and vegetables and other fruits and vegetables is necessary for ensuring minimum dietary diversity (MDD) [26,27,28]. But unfortunately, only less than one-third (29%) of children’s (aged 6–23 months) intake practices can meet the WHO standard of MDD globally [29]. For example, in India itself, only 16% of children can meet the global standard of nutritional adequacy, which is even lower than in African countries such as Mali (22%), Ghana (26%), and Tanzania (21%) [30]. Consequently, poor nutritional status, either directly or indirectly, eventually ends in 60% casualties out of 10.9 million under-five deaths [31]. Likewise any other developing country, the situation remains almost the same in the Indian context, with a meager share of children with adequate dietary diversity and an associated 40% prevalence of chronic undernutrition [32] and high under-five mortality [15]. In particular, micronutrient inadequacy through poor dietary intake not only deteriorates optimal growth and development but also elevates life risk by predisposing children to opportunistic infections and illnesses [15]. Therefore dietary diversity, an important proxy of nutritional adequacy [22], is inevitable for optimal health since it elevates the micronutrient density of complementary foods that can further nourish optimal growth and development among young children. To prevent the issue of malnutrition in the nation, the government is implementing Integrated Child Development Services Scheme (ICDS) and Anganwadi Services as targeted interventions. The Ministry of Women and Child Development is encouraging the establishment of Poshan Vatikas (Nutri-gardens) across all Anganwadi centers in order to provide a fresh supply of fruits, vegetables, and even medicinal plants so that multiple goals of dietary diversity, food security, and malnutrition control could be achieved.

Although the pattern of dietary diversity [32,33], underlying determinants [34], and the association between nutritional status and the same [35] among children aged 6–23 months have already been studied in the Indian context, no study has demonstrated a clear association between dietary diversity scores and the three forms of undernourishment and severe undernourishment. Child undernourishment among children aged 6–23 months is an imperative alarm for the public health authorities in India. Goal 2 of the 17 Sustainable Development Goals (SDGs) is to eradicate all forms of hunger by 2030. The most disadvantaged group may have a greater risk of undernutrition and poor health as they cannot access safe, nutritious, affordable, and sustainable diets or food (Target 2.1). So, it is necessary to determine the effect of dietary diversity on child growth outcomes. The SDG-3 is ‘Good Health and Well-being’, which is essential for improving child growth conditions. Therefore, the present study fills this gap by finding out existing dietary diversity and its associated role in child growth outcomes so that policymakers can bring target groups into the focal area of policy implications for uniform coverage of attainment of MDD throughout the nation.

## 2. Methods

### 2.1. Study Design and Sample

This is the secondary data analysis from data of the fourth round of the NFHS conducted in 2015–2016, a cross-sectional national representative survey to estimate the effects of dietary diversity on growth outcomes among children aged 6–23 months. The NFHS-4, 2015–2016 was conducted under the Ministry of Health and Family Welfare (MoHFW) of India, with the coordination and technical guidance provided by the International Institute of Population Science (IIPS), Mumbai. The nationwide representative survey of India includes vital information about women, family planning, and child health. This NFHS-4 collected data from a population-representative sample of 699,686 women aged 15–49 years and 112,122 men aged 15–54 surveyed from 601,509 households [36]. The response rate for women and men were 97% and 92%, respectively. According to a list provided by municipal corporation offices, this sample was selected using a stratified sampling strategy consisting of 28,586 clusters (20,509 in rural, 8397 in urban, and 130 from slums). More details about the sampling methodology were provided in the NFHS-4 report of India.

### 2.2. Study Participants

**Figure 1:**
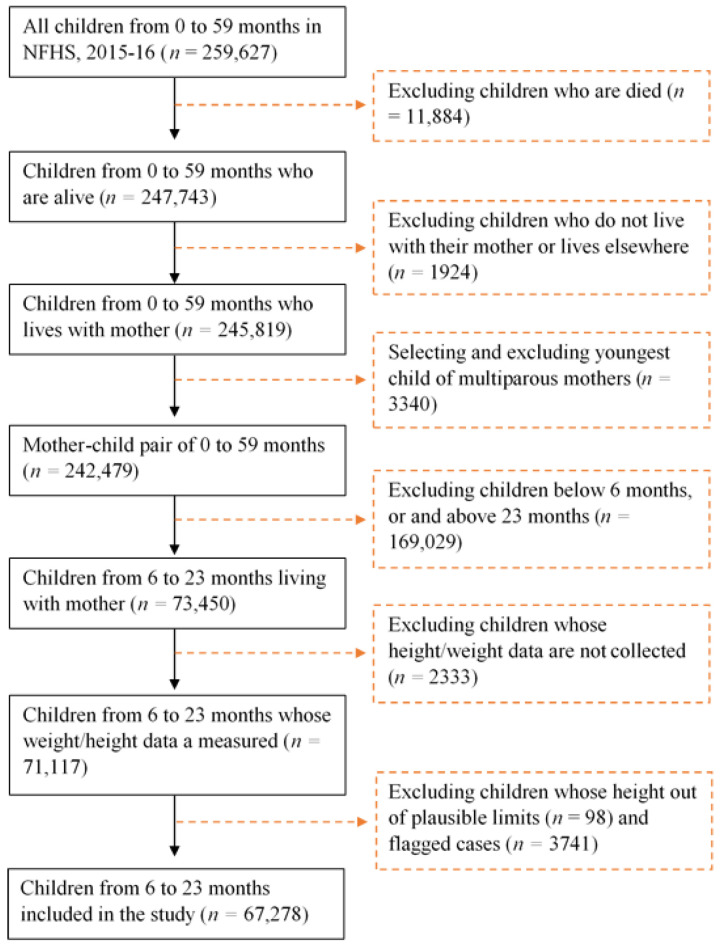
Flow diagram showing children of 6 to 23 months included in the study for analyses from NFHS-4, 2015–2016, India. NFHS = National Family Health Survey.

A total number of 259,627 children aged between 0 and 59 months were born in the last five years (*n* = 259,627). Out of these, 192,349 children were excluded. Among them, 11,884 children have died, excluding those children who did not live with their mother or lived elsewhere (*n* = 1924), selecting and excluding those youngest children of multiparous mothers (*n* = 3340), excluding children whose ages are below six months, or and above 23 months (*n* = 169,029), excluding children those height/weight data are not collected (*n* = 2333) (Figure 1). Children whose height is out of plausible limits (*n* = 98) and flagged cases (*n* = 3741) were excluded. Finally, 67,278 children aged between 6 to 23 months were selected for this study (Figure 1).

### 2.3. Growth Outcomes Calculation

For this study, the children’s weight and height were measured directly to create the ethnographic variables. The Seca 874 digital scale and Seca 417 infantometer measured the weight and height of children aged 6–23 months, respectively, and specially trained health investigators collected the measurements [36]. Training videos in English and Hindi were created to outline the proper procedures for taking height and weight measurements and demonstrate how to do so in detail to ensure that measurements were taken correctly and consistently [36]. Based on WHO standards, stunting, wasting, and underweight were calculated from height-for-age Z-scores (HAZ), weight-for-height Z-scores (WHZ), and weight-for-age Z-scores (WAZ) [37]. Stunting, as measured by a children’s height in relation to their age, is a sign of chronic undernutrition caused by long-term food deprivation or illness. Weight-for-height Z-scores (wasting) assess acute undernutrition due to more recent food deprivation and disease. WAZ is a composite measure that reflects acute and chronic nutrients, although it cannot distinguish between them [38]. Weight-for-age Z-scores assess a child’s body mass in relation to his or her chronological age and act as a proxy for underweight. Children whose HAZ, WHZ, and WAZ were below minus two standard deviations (−2 SD) from the reference population’s median were considered stunted, wasted, and underweight, respectively. Three standard deviations (−3 SD) from the reference population’s median were considered severely stunted, severely wasted, and severely underweight, respectively.

### 2.4. Dietary Diversity Calculation

The dietary diversity score (DDS) was defined as the number of groups consumed by the children the day before [39]. To analyze the DDS among children aged 6–23 months, we adopted the WHO’s IYCF guidelines as internationally recognized supplementary feeding guidelines tools [40]. This analysis contains information on food items in the dataset used to calculate dietary diversity scores. The NFHS-4 survey collected data on food items based on the number of food groups consumed by a child over the last 24 h. Trained health investigators collected the information from the mothers. The information from the mothers was collected using structured questionnaires through Computer Assisted Personal Interviewing (CAPI) method. Based on the WHO’s IYCF guidelines, these foods were initially classified into seven major food groups. These food groups are (i) grains, roots, and tubers; (ii) legumes and nuts; (iii) dairy products (milk, yogurt, and cheese); (iv) flesh foods (meat, fish, poultry, and liver/organ meats); (v) eggs; (vi) vitamin A-rich fruits and vegetables; (vii) other fruits and vegetables [28]. The DDS was constructed based on the information on food consumption (e.g., if a child consumed at least one food item from a food group throughout the day before, the group was assigned a value of one (1) for that child, and zero (0) if not consumed). Then the group scores are summed up to obtain the DDS, which ranges from 0 to 7, where 0 represents the non-consumption of any food items in the food groups, and 7 represents the highest level of varied foods or diet diversification. MDD was diagnosed if a child consumed four or more food groups (MDD ≥ 4) out of the seven food groups the day before.

### 2.5. Other Covariates

Other explanatory variables or covariates were selected based on their significant relationship with childhood undernutrition from reviewed literature and the study’s objective, as well as their availability in the Demographic Health Surveys (DHS) dataset. The variables consisted of the characteristics of the child reported by the mother in this study: the sex of the child (male and female), the child’s birthplace comprised into three categories (at home, institution/health facility, and other places), size of the child at birth (larger, average, smaller, and not reported), currently breastfeeding (no and yes), and at last any presence of diarrhea and fever in the past two weeks before the interview were categorized into the following ways: yes, no, and not reported. The mother’s estimate of the child’s size at birth is subjective but can be a useful proxy for the child’s weight.

### 2.6. Statistical Analyses

This study presented categorical variables as proportions and continuous variables as means and standard deviation (Mean ± SD). Bivariate and multivariable analyses were used to identify the association between dietary diversity and growth outcomes (i.e., stunting, wasting, and being underweight). Descriptive statistics are also performed to analyze the data. This study calculated the percentage of stunting, wasting, underweight and severely stunting, severe wasting, and severely underweight children. Secondly, descriptive statistics were performed to estimate the frequency and percentage of the study variable. The DDS was considered a continuous and independent variable of interest by counting the food groups children consumed the previous day before the survey. We created a separate model for each anthropometric outcome, using the dietary diversity score as an independent variable. The same was performed for the MDD as a definite indicator as well. Statistical analyses were conducted by entering each food group into a bivariate and multivariable model to test its associations with the growth outcomes of the children. The multivariable logistic regression model was used to calculate the crude odds ratio (COR) and adjusted odds ratio (AOR) with confidence intervals (CIs) of 95% and considered results significant if the *p*-value was less than 0.05 (*p* < 0.05). The sex of the child, size of the child at birth, presence of fever (yes/no), and diarrhea (yes/no) during the two weeks before the survey were controlled in the multivariable models. All of the analyses were performed in Stata 17 version (StataCorp L.P., College Station, TX, USA) and graphically visualized in Excel (Microsoft Corporation, Redmond, WA, USA).

## 3. Results

### 3.1. Characteristics of the Children Aged 6–23 Months

Table 1 represents the general characteristics of the children aged 6–23 months included in this study. Out of 24,157 children were found as stunting (HAZ < −2 SD), 15,254 children were wasting (WHZ < −2 SD), and 21,325 children were seen as underweight (WAZ < −2 SD), and the rest of the children were considered as normal Nutrition (Table 1). Similarly, out of the total dataset, 10,954 children were found as severely stunting (HAZ < −3 SD), 5601 children were severely wasting (WHZ < −3 SD), and 6881 children were severely underweight (WAZ < −3 SD). Of them, 51.9% and 48.1% were male and female. Their mean (SD) age was 14.4 (5.2) months; about 82.2% were born in health facilities and 17.6% at home. Among the studied children, 35.9% and 16.2% were stunted and severe stunting; 23.8% and 8.5% were wasting and severe wasting; more than 32% and 10% were underweight and severely underweight, respectively. Approximately 68% of children had an average size at birth, and nearly 85% of children were currently involved in breastfeeding. More than 85% of children had no diarrhea within two weeks before the survey, and 82.4% had no sign of fever.

### 3.2. Dietary Diversity of Children Aged 6 to 23 Months in India

**Figure 2:**
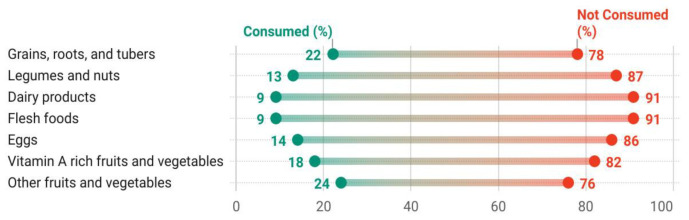
The proportion of children 6–23 months consumed different types of food.

**Figure 3:**
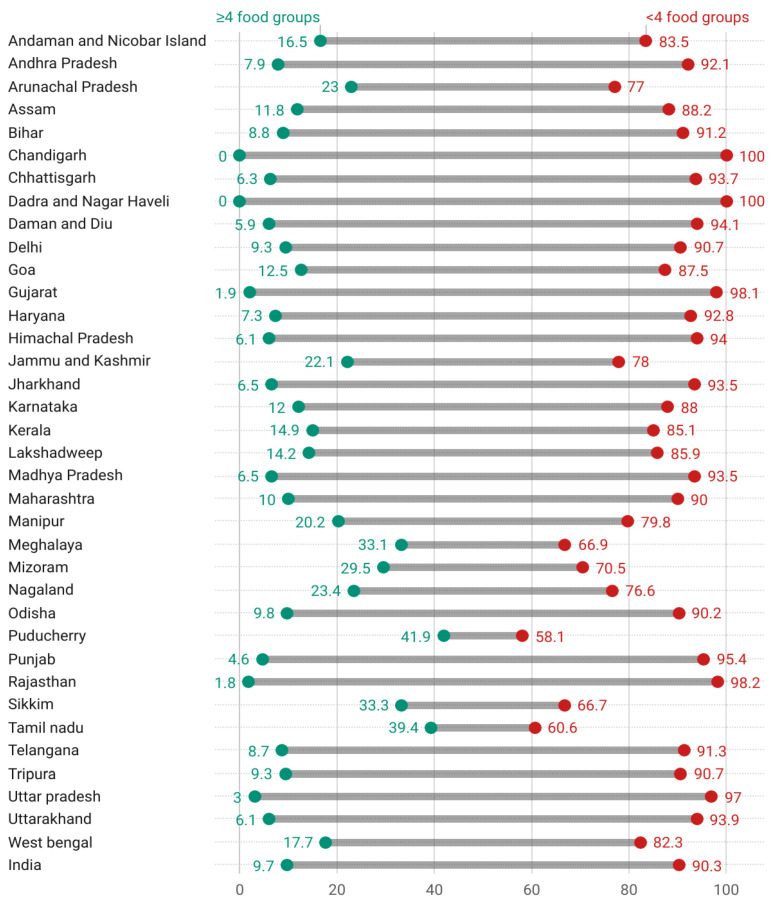
State and UTs wise proportion of children 6–23 months consumed food (≥4 food groups vs. <4 food groups) in India. UTs = Union Territories.

Figure 2 presents the proportion of children in food groups consumed on the previous day before the survey. This analysis found that most children in India’s states and union territories did not meet the recommended MDD of more than four food groups the day before. Except for a few states, less than 25% of children across India had received a minimum diversified diet (Figure 3). Grains, roots, tubers (22%), and other fruits and vegetables (24%) were the most commonly consumed foods. The remaining food groups, including legumes and nuts (13%), dairy products (9%), flesh foods (9%), as well as eggs (14%), and vitamin A-rich foods and vegetables (18%), were reported as a lesser proportion of foods consumed by the children.

### 3.3. Association between Dietary Diversity and Undernutrition

Table 2 analyses the results of an association between dietary diversity and stunting, wasting, and underweight among children aged 6–23 months in India. Based on the multivariable analysis using the DDS and MDD as independent variables, varied food intake was significantly associated with reducing stunting, wasting, and being underweight for children. The possibility of being stunted, wasting, and underweight among children aged 6–23 months decreased significantly as the number of food groups consumed increased. Consequently, the analysis of dietary diversity scores and MDD reported that children who consumed a diverse diet or varied foods are less likely to be undernourished than those with a less diverse diet. The odds of being stunted (AOR = 1.23; 95% CI; 1.17–1.30), wasted (AOR = 1.29; 95% CI; 1.21–1.38), and underweight (AOR = 1.47; 95% CI; 1.39–1.56) were more than one time higher among children aged 6–23 months who did not receive the MDD (<4 food groups) compare to those obtained the MDD (≥4 food groups).

### 3.4. Association between Dietary Diversity and Severe Undernutrition

A similar result in Table 3 presents the analysis of the association between dietary diversity and severe stunting, wasting, and being underweight among children aged 6–23 months in India. Here the probability of being stunted was 1.2 times (AOR = 1.27; 95% CI; 1.18–1.36), wasting 1.2 times (AOR = 1.24; 95% CI; 1.12–1.37), and underweight 1.4 times (AOR = 1.44, 95% CI; 1.31–1.58) higher among children who consumed less than four food groups than children who intake more diverse food (MDD ≥4 food groups).

### 3.5. Association between Specific Food Groups and Undernutrition

Table 4 shows the unadjusted and adjusted odds from multivariable logistic regression models for the association between specific food groups and stunting, wasting, and underweight among children 6–23 months in India. The consumption of dairy products, flesh foods, eggs, and other fruits and vegetables has been significantly associated with children’s stunting. Children who did not intake any dairy products (AOR = 1.13; 95% CI; 1.08–1.22), flesh foods (AOR = 1.14; 95% CI; 1.07–1.21), eggs (AOR = 1.13; 95% CI; 1.07–1.2), and other fruits and vegetables (AOR = 1.12; 95% CI; 1.07–1.17) have a higher probability of becoming stunted in the adjusted model. However, children who did not consume grains, roots, and tubers were at a lower risk of stunted (AOR = 0.92; 95% CI; 0.88–0.96).

On the other hand, the risk of being wasted was more than one time higher among children who did not consume legumes and nuts (AOR = 1.07; 95% CI; 1–1.14), dairy products (AOR = 1.14; 95% CI; 1.06–1.23), flesh foods (AOR = 1.18; 95% CI; 1.08–1.27), eggs (AOR = 1.13; 95% CI; 1.05–1.2), and other fruits and vegetables (AOR = 1.1; 95% CI; 1.04–1.16). A significant association was also observed between the vitamin A-rich fruits and vegetables, and wasting depicts that the children who did not consume any Vitamin A-rich fruits and vegetables were at a lower probability of becoming wasted in the adjusted model (AOR = 0.93; 95% CI; 0.88–0.98) (Table 4).

Moreover, the analysis shows that children who did not consume any dairy products (AOR = 1.23; 95% CI; 1.16–1.32), flesh foods (AOR = 1.24; 95% CI; 1.16–1.33), eggs (AOR = 1.3; 95% CI; 1.18–1.43) and other fruits, and vegetables (AOR = 1.20; 95% CI; 1.12–1.29) were more than one times higher probability of being underweight than the children who consumed these products. However, the likelihood of becoming underweight for children who did not consume any grains, roots, and tubers (AOR = 0.95; 95% CI; 0.91–0.99) and vitamin A-rich fruits and vegetables (AOR = 0.93; 95% CI; 0.89–0.98) is lower compared to children who do (Table 4).

### 3.6. Association between Specific Food Groups and Severe Undernutrition

Table 5 presents the multivariable logistic regression analysis of the association between food groups and severe stunting, severe wasting, and severe underweight among children aged 6–23 months in India. Severely stunting was more than one time higher among children who did not consume dairy products (AOR = 1.16; 95% CI; 1.07–1.26), eggs (AOR = 1.19; 95% CI; 1.10–1.28) and other fruits, and vegetables (AOR = 1.1; 95% CI; 1.04–1.17) compared the children who consumed. However, in the previous table, a similar association is also observed between grains, roots, and tubers and several stunted children. Children were at a lower risk of being severely stunted than those who consumed grains, roots, and tubers (AOR = 0.93; 95% CI; 0.89–0.99).

Children who did not consume any legumes and nuts (AOR = 1.12; 95% CI; 1.01–1.23), dairy products (AOR = 1.15; 95% CI; 1.03–1.29), and flesh foods (AOR = 1.17; 95% CI; 1.03–1.32) had a higher likelihood of becoming severely wasted. The diverse diet foods such as grains, roots, tubers, and vitamin A-rich fruits and vegetables were at a lower risk of being severely wasted among children who did not consume these. However, this association was not statistically significant in this adjusted model (Table 4).

Furthermore, the odds of becoming severely underweight were 1.2 times higher among children who did not consume dairy products (AOR = 1.24; 95% CI; 1.12–1.38) compared to the children who intake. Similarly, children who did not consume any flesh foods (AOR = 1.18; 95% CI; 1.06–1.31), eggs (AOR = 1.3; 95% CI; 1.18–1.43) and other fruits, and vegetables (AOR = 1.2; 95% CI; 1.12–1.29) had a higher likelihood of becoming severely underweight in this logistic regression model. However, the likelihood of becoming severely underweight for children who did not consume any Vitamins A-rich fruits and vegetables is as lower as compared to the children who consumed vitamin A-rich foods (AOR = 0.92; 95% CI; 0.85–0.99), and this association was statistically significant in the adjusted table (Table 4).

## 4. Discussion

The present study examines the role of dietary diversity intake and its impact on growth outcomes among children aged 6–23 months in India. The findings revealed that stunting as the prevalent form of both stunting (35.9%) and severe stunting (16.2%), followed by underweight (WAZ < −2 SD: 32.8%, WAZ < −3 SD: 10.4%) and wasting (WHZ < −2 SD: 23.8% WHZ < −3 SD: 8.5%) prevailed among children who are similar to the previous study in India [41]. In this regard, inadequate intake of a diversified diet was found to be the predisposing factor of all forms of undernutrition except stunting and severe undernutrition, viz., stunting, wasting, and underweight. In particular, many prior studies in India [41] and abroad [22,42,43] have already exemplified that diverse dietary intake is positively associated with optimal nutritional status since dietary diversity elevates micronutrient density and aid in proper nourishment.

The previous studies did not find a significant association between stunting and dietary diversity [44]. This was consistent with our findings that dietary diversity had an insignificant association with stunting. Still, it did have a significant association with severe stunting, with a two-fold increase in prevalence (12%) among children aged 6–23 months. The possible reason for this variation could be that inadequate intake of MDD causes a poor immune system due to micronutrient deficiencies. Also, when it is subjected to severe stunting, chronic nutritional deficiency and exposure to diseases due to unhygienic households further elevate the risk of severe stunting among children. Contrarily wasting and being underweight, along with their respective severe forms, were significantly associated with dietary diversity and were in tune with several findings [22,45]. Nevertheless, none of them did mirror the association between severe forms of undernutrition and dietary diversity. With this backdrop, the pattern of dietary intake also played a crucial role in growth outcomes among children since.

The present study found that unlike animal-source food (dairy, flesh, egg), the consumption of plant-source foods was prevalent among children aged 6–23 months in India. Although almost a quarter of children rely on grains, fruits, and veggies, only one-third of children can receive MDD in Puducherry, Tamil Nadu, Sikkim, and the North-Eastern states. Except for Assam and Tripura, Chandigarh, Dadra, Nagar Haveli, Punjab, and empowered action groups (EAG) states were the worst performing states with the lowest percentage of children with MDD. Such interregional variation of dietary intake could be justified on the ground of the socio-economic development divide between EAG and Non-EAG states, where EAG states were more prone to poor literacy level, household wealth status, and less aware of the necessities of balanced and diverse diets within the initial years of life than Non-EAG fellow [46]. A recent national report on nutrition [47] revealed that EAG states hold more than two-fifths of stunting, wasting, and underweight cases, nearly double that of two Non-EAG states (i.e., Goa and Jammu-and-Kashmir).

A noteworthy finding of the current study was that the pattern of dietary intake also played a crucial role in growth outcomes among children. Against this backdrop, consuming animal-source foods, such as dairy products, flesh, and eggs, was significantly associated with decreased risk of stunting and severe stunting. This is in agreement with an Indonesian [48] study finding which revealed animal source food is rich in micronutrients due to its large content of iron, vitamin A, vitamin B-12, zinc, iodine, and protein which could hardly be availed in adequate quantity from plant source foods. Consequently, inadequate intake of animal-source foods might elevate the risk of stunting among children [49]. Likewise, consuming animal-source food again had a crucial interplay with decreasing the risk of wasting and being underweight and their respective severe forms. It is consistent with Iranian [50] and Cambodian [51] studies as well.

In particular, flesh food was found to be a crucial protective factor against both wasting, and severe wasting among children since including flesh in dietary intake reduces the risk of wasting and severe wasting by 56% among children aged 6–23 months. At the same time, a possible explanation for this lies in the role of the bioavailability of protein in flesh foods other than any non-flesh foods, which hardly contain 20–60% protein density of flesh food [52]. Besides, the intake of dairy products also exemplified a negative association with wasting and severe form among children, which is consistent with another Indian study [53]. While this might be due to its high nutritional value with large content of calcium, vitamin A, and vitamin B-12, it can be consumed daily due to its availability throughout the year [54]. In addition, the intake of dairy products, meat, and eggs had a crucial interplay with decreased risk of being underweight and along with severe form and was corroborated by another study’s findings [51,53]. Nevertheless, none of the studies incorporated severe forms of being underweight.

The present study has several policy implications. First, area-specific intervention is of utmost importance to address undernourishment across the worst-performing states by ensuring dietary intake with a basket of diversified diets among children in their initial years. Second, it is also essential to ensure the dietary intake with a big basket of nutrient-rich diets among children. Third, awareness-generating programs are needed to create awareness among mothers or caregivers regarding the nutritional value of animal-source food in averting malnutrition among children. Moreover, policymakers should bring the underprivileged children into the focal area of policy implication by providing either direct cash incentives or food to prevent long-term malnutrition by customizing food insecurity among the needy.

### Limitations of the Study

Despite several policy implications, the current study should be considered in light of its shortcomings. Firstly, the study findings are subjected to recall bias since the study incorporates self-reporting data of 24-h maternal recall of dietary intake. Secondly, the study only includes surviving children, most of whom were socio-economically privileged during the survey period. Therefore, the study findings are prone to survival bias and unable to capture the actual estimates. Thirdly, the present study cannot capture the causal relationship between growth outcomes and dietary diversity due to the cross-sectional nature of the data. In fact, unlike dietary diversity, there are other determinants, such as duration of breastfeeding, food custom, and indoor hygiene status, which have not been included in the current study. Therefore, future longitudinal studies can be undertaken to determine the causality between diversified dietary intake and growth outcomes among children.

## 5. Conclusions

In India, the colossal share of pediatric malnutrition raises questions on the extent of grass root level implications of nutritional programs, especially among underprivileged children. In this regard, the present study affirms that feeding children with a nutrient-dense diet are inevitable (beyond filling the stomach with diversified diets to boost both nutritional status and optimal growth among children). Indeed, inadequate intake of dietary diversity has a direct bearing on growth outcomes in the form of wasting and being underweight along with respective severe forms among children. Nevertheless, children with adequate intake of animal-source food such as dairy products, flesh, and eggs are least exposed to stunting, wasting, and being underweight. Local community health workers (CHWs), including Anganwadi workers (AWWs), and auxiliary nurse-midwife (ANM), should be trained firmly since they act as synchronizing medium between community pediatric needs and utilization of healthcare programs by enhancing knowledge and awareness regarding the necessities of dietary diversity and nutritional values in the Indian context, where the majority of children reside in a rural area with poorly educated mothers. The currently available interventions are the Integrated Child Development Services (ICDS) scheme, Mid-Day Meal Scheme, and nutrition programs by digitizing the Anganwadis to address critical nutrition-sensitive issues and promote optimal nutrition for 6–23 months. Moreover, government and non-governmental organizations (NGOs) must come forward and synchronize for a committed and coordinated leadership in averting pediatric malnutrition and associated morbidities and mortalities.

## Figures and Tables

**Table 1:** Descriptive characteristics of the children aged 6–23 months included in the study, NFHS, 2015–2016 (*n* = 67,278).

**Characteristics**	**Mean (SD) and Frequency (%)**	
**Age of Children in Months**	**14.4 (SD = 5.2 Months)**	
Growth outcomes		
Prevalence of undernutrition		
Stunting (HAZ < −2 SD)	35.9	24,157
Wasting (WHZ < −2 SD)	23.8	15,254
Underweight (WAZ < −2 SD)	32.8	21,325
Prevalence of severe undernutrition		
Severely stunting (HAZ < −3 SD)	16.2	10,954
Severely wasting (WHZ < −3 SD)	8.5	5601
Severely underweight (WAZ < −3 SD)	10.4	6881
Sex of Child		
Male	51.9	34,915
Female	48.1	32,363
Child’s birthplace		
At Home	17.6	13,908
Institution/health facility	82.2	53,199
Other places	0.2	171
Size of the child at birth		
Larger	19.6	11,617
Average	67.5	46,557
Smaller	11.8	7940
Not reported	1.2	1164
Currently breastfeeding		
No	15.2	9598
Yes	84.8	57,680
Diarrhea in the last 2 weeks		
Yes	14.7	9666
No	85.3	57,562
Not reported	0.06	50
Fever in last 2 weeks		
Yes	17.5	11,721
No	82.4	55,522
Not reported	0.04	35

NFHS = National Family Health Survey, SD = standard deviation, HAZ = height-for-age Z-scores, WHZ = weight-for-height Z-scores, WAZ = weight-for-age Z-scores.

**Table 2:** Bivariate and multivariable logistic regression analysis of the association between dietary diversity and stunting, wasting, and underweight among children aged 6–23 months in India.

**Dietary Diversity Score (DDS)**	**Growth Outcomes**								
	**Stunting (HAZ < −2 SD)**			**Wasting (WHZ < −2 SD)**			**Underweight (WAZ < −2 SD)**		
	**Frequency (%)**	**OR** **(95% CI)**	**AOR ^a^** **(95% CI)**	**Frequency (%)**	**OR** **(95% CI)**	**AOR ^a^** **(95% CI)**	**Frequency (%)**	**OR** **(95% CI)**	**AOR ^a^** **(95% CI)**
Score 0	12,460 (34.8)	^®^	^®^	8764 (25.5)	^®^	^®^	11,903 (34.2)	^®^	^®^
Score 1	4904 (38.2)	1.2 *** (1.05–1.15)	0.92 *** (0.88–0.96)	2801 (22.6)	0.83 *** (0.79–0.87)	0.86 *** (0.82–0.91)	4157 (33.4)	0.92 *** (0.88–0.96)	0.85 *** (0.81–0.89)
Score 2	2791 (38.4)	1.09 *** (1.04–1.15)	0.88 *** (0.84–0.93)	1542 (22.3)	0.79 *** (0.74–0.84)	0.83 *** (0.78–0.89)	2268 (32.6)	0.86 *** (0.81–0.90)	0.78 *** (0.74–0.83)
Score 3	1577 (35.6)	1.02 (0.95–1.09)	0.80 *** (0.75–0.86)	896 (20.5)	0.77 *** (0.71–0.83)	0.83 *** (0.77–0.89)	1236 (27.6)	0.76 *** (0.71–0.82)	0.70 *** (0.65–0.75)
Score 4	946 (37.2)	1.06 (0.98–1.16)	0.84 *** (0.77–0.91)	487 (20.8)	0.71 *** (0.64–0.78)	0.75 *** (0.68–0.84)	707 (29.5)	0.74 *** (0.68–0.81)	0.68 *** (0.62–0.74)
Score 5	613 (33.2)	0.99 (0.89–1.09)	0.76 *** (0.68–0.84)	301 (20.4)	0.63 *** (0.55–0.71)	0.68 *** (0.60–0.78)	427 (26.0)	0.63 *** (0.57–0.71)	0.57 *** (0.52–0.64)
Score 6	378 (34)	0.93 (0.82–1.05)	0.71 *** (0.63–0.81)	185 (17.3)	0.59 *** (0.51–0.70)	0.66 *** (0.56–0.77)	290 (29.4)	0.68 *** (0.60–0.78)	0.63 *** (0.55–0.72)
Score 7	447 (33.9)	0.9 * (0.80–1.01)	0.69 *** (0.62–0.79)	252 (22.6)	0.69 *** (0.6–0.76)	0.76 *** (0.66–0.87)	306 (23.6)	0.57 *** (0.50–0.65)	0.53 *** (0.46–0.60)
**Minimum dietary diversity (MDD)**	**Frequency (%)**	**OR** **(95% CI)**	**AOR ^a^** **(95% CI)**	**Frequency (%)**	**OR** **(95% CI)**	**AOR ^a^** **(95% CI)**	**Frequency (%)**	**OR** **(95% CI)**	**AOR ^a^** **(95% CI)**
≥4 food groups	2384 (35.1)	^®^	^®^	1225 (20.4)	^®^	^®^	1730 (27.6)	^®^	^®^
<4 food groups	21,732 (36)	1.05 * (0.99–1.10)	1.23 *** (1.17–1.30)	14,003 (24.1)	1.38 *** (1.29–1.47)	1.29 *** (1.21–1.38)	19,564 (33.3)	1.41 *** (1.33–1.5)	1.47 *** (1.39–1.56)

*** *p* < 0.01, * *p* < 0.1; ^a^ Adjusted for age of children, sex of child, currently breastfeeding, diarrhea and fever in last 2 weeks; CI = Confidence Interval in parentheses, OR = Odds Ratio, AOR = Adjusted Odds Ratio, ^®^ = Reference category, HAZ = Height-for-Age Z-scores, WHZ = Weight-for-Height Z-scores, WAZ = Weight-for-Age Z-scores. Note: Both models fit the data equally well (all *p* > 0.10 in the likelihood ratio test).

**Table 3:** Bivariate and multivariable logistic regression analysis of the association between dietary diversity and severe stunting, severe wasting, and severely underweight among children aged 6–23 months in India.

**Dietary Diversity Score (DDS)**	**Growth Outcomes**								
	**Severely Stunting (HAZ < −3 SD)**			**Severely Wasting (WHZ < −3 SD)**			**Severely Underweight (WAZ < −3 SD)**		
	**Frequency (%)**	**OR** **(95% CI)**	**AOR ^a^** **(95% CI**	**Frequency (%)**	**OR** **(95% CI)**	**AOR ^a^** **(95% CI)**	**Frequency (%)**	**OR** **(95% CI)**	**AOR ^a^** **(95% CI**
Score 0	5808 (16)	^®^	^®^	3295 (9.5)	^®^	^®^	3918 (11.1)	^®^	^®^
Score 1	2189 (17.2)	1.02 (0.97–1.08)	0.89 *** (0.84–0.94)	989 (7.7)	0.79 *** (0.74–0.86)	0.84 *** (0.78–0.91)	1272 (10)	0.85 *** (0.79–0.91)	0.79 *** (0.74–0.85)
Score 2	1262 (17.2)	1.03 (0.97–1.10)	0.87 *** (0.81–0.93)	532 (7.3)	0.75 *** (0.68–0.82)	0.8 *** (0.72–0.88)	681 (10.1)	0.79 *** (0.72–0.86)	0.76 *** (0.67–0.79)
Score 3	661 (14.9)	0.89 ** (0.82–0.98)	0.74 *** (0.69–0.81)	337 (7.1)	0.80 *** (0.71–0.9)	0.87 ** (0.78–0.98)	405 (8.8)	0.79 *** (0.71–0.88)	0.73 *** (0.69–0.82)
Score 4	410 (16.6)	0.96 (0.86–1.07)	0.79 *** (0.71–0.89)	196 (8.2)	0.80 *** (0.69–0.93)	0.87 * (0.75–1.01)	223 (8.8)	0.75 *** (0.65–0.86)	0.69 *** (0.6–0.8)
Score 5	254 (14)	0.86 ** (0.75–0.99)	0.7 *** (0.61–0.8)	98 (5.4)	0.58 *** (0.47–0.71)	0.64 *** (0.52–0.79)	130 (8.3)	0.63 *** (0.53–0.76)	0.58 *** (0.48–0.7)
Score 6	154 (12.1)	0.803 ** (0.68–0.95)	0.655 *** (0.55–0.78)	65 (5.2)	0.59 *** (0.46–0.77)	0.67 *** (0.52–0.86)	73 (6.5)	0.55 *** (0.43–0.69)	0.51 *** (0.40–0.65)
Score 7	199 (16.2)	0.87 * (0.75–1.02)	0.72 *** (0.62–0.84)	84 (7)	0.64 *** (0.51–0.8)	0.72 *** (0.57–0.9)	104 (8.6)	0.65 *** (0.53–0.8)	0.61 *** (0.5–0.75)
**Minimum dietary diversity (MDD)**	**Frequency (%)**	**OR** **(95% CI)**	**AOR ^a^** **(95% CI)**	**Frequency (%)**	**OR** **(95% CI)**	**AOR ^a^** **(95% CI**	**Frequency (%)**	**OR** **(95% CI)**	**AOR ^a^** **(95% CI**
≥4 food groups	1017 (15.1)	^®^	^®^	443 (6.7)	^®^	^®^	530 (8.3)	^®^	^®^
< 4 food groups	9920 (16.3)	1.12 *** (1.05–1.2)	1.27 *** (1.18–1.36)	5153 (8.7)	1.34 *** (1.22–1.49)	1.24 *** (1.12–1.37)	6339 (10.6)	1.39 *** (1.27–1.53)	1.44 *** (1.31–1.58)

*** *p* < 0.01, ** *p* < 0.05, * *p* < 0.1; ^a^ Adjusted for age of children, sex of child, currently breastfeeding, diarrhea and fever in last 2 weeks; CI = Confidence Interval in parentheses, OR = Odds Ratio, AOR = Adjusted Odds Ratio, ^®^ = Reference category, HAZ = Height-for-Age Z-scores, WHZ = Weight-for-Height Z-scores, WAZ = Weight-for-Age Z-scores. Note: Both models fit the data equally well (all *p* > 0.10 in the likelihood ratio test).

**Table 4:** Multivariable logistic regression analysis of the association between food groups and stunting, wasting, and underweight among children aged 6–23 months in India.

Characteristics	Growth Outcomes					
	Stunting (HAZ < −2 SD)		Wasting (WHZ < −2 SD)		Underweight (WAZ < −2 SD)	
	Crude OR (95% CI)	Model ^a^ AOR (95% CI)	Crude OR (95% CI)	Model ^a^ AOR (95% CI)	Crude OR (95% CI)	Model ^a^ AOR (95% CI)
Food Groups Grains, roots, and tubers						
Yes	^®^	^®^	^®^	^®^	^®^	^®^
No	0.87 *** (0.84–0.91)	0.92 *** (0.88–0.96)	1.16 *** (1.11–1.22)	1 (0.95–1.05)	1.06 *** (1.02–1.10)	0.95 ** (0.91–0.99)
Legumes and nuts						
Yes	^®^	^®^	^®^	^®^	^®^	^®^
No	0.99 (0.95–1.04)	1 (0.95–1.06)	1.29 *** (1.22–1.36)	1.07 ** (1–1.14)	1.25 *** (1.19–1.31)	1.06 * (1–1.12)
Dairy products						
Yes	^®^	^®^	^®^	^®^	^®^	^®^
No	1.13 *** (1.07–1.19)	1.15 *** (1.08–1.22)	1.37 *** (1.28–1.46)	1.14 *** (1.06–1.23)	1.46 *** (1.38–1.55)	1.23 *** (1.16–1.32)
Flesh foods						
Yes	^®^	^®^	^®^	^®^	^®^	^®^
No	1.11 *** (1.05–1.16)	1.14 *** (1.07–1.21)	1.44 *** (1.35–1.54)	1.18 *** (1.08–1.27)	1.5 *** (1.42–1.59)	1.24 *** (1.16–1.33)
Eggs						
Yes	^®^	^®^	^®^	^®^	^®^	^®^
No	1.08 *** (1.04–1.13)	1.13 *** (1.07–1.2)	1.37 *** (1.3–1.45)	1.13 *** (1.05–1.2)	1.44 *** (1.37–1.52)	1.24 *** (1.17–1.32)
Vitamin A-rich fruits and vegetables						
Yes	^®^	^®^	^®^	^®^	^®^	^®^
No	0.97 (0.9–1.01)	1 (0.95–1.05)	1.15 *** (1.1–1.20)	0.93 *** (0.88–0.98)	1.13 *** (1.08–1.18)	0.93 *** (0.89–0.98)
Other fruits and vegetables						
Yes	^®^	^®^	^®^	^®^	^®^	^®^
No	1.02 (0.98–1.06)	1.12 *** (1.07–1.17)	1.26 *** (1.21–1.32)	1.1 *** (1.04–1.16)	1.29 *** (1.24–1.34)	1.20 *** (1.15–1.26)

*** *p* < 0.01, ** *p* < 0.05, * *p* < 0.1; ^a^ Adjusted for age of children, sex of the child, currently breastfeeding, diarrhea and fever in last 2 weeks; CI = Confidence Interval in parentheses, OR = Odds Ratio, AOR = Adjusted Odds Ratio, ^®^ = Reference category, HAZ = Height-for-Age Z-scores, WHZ = Weight-for-Height Z-scores, WAZ = Weight-for-Age Z-scores.

**Table 5:** Multivariable logistic regression analysis of the association between food groups and severe stunting, severe wasting, and severely underweight among children aged 6–23 months in India.

Characteristics	Growth Outcomes					
	Severely Stunting (HAZ < −3 SD)		Severely Wasting (WHZ < −3 SD)		Severely Underweight (WAZ < −3 SD)	
	Crude OR (95% CI)	Model ^a^ AOR (95% CI)	Crude OR (95% CI)	Model ^a^ AOR (95% CI)	Crude OR (95% CI)	Model ^a^ AOR (95% CI)
Food groups						
Grains, roots, and tubers						
Yes	^®^	^®^	^®^	^®^	^®^	^®^
No	0.93 *** (0.89–0.98)	0.93 ** (0.89–0.99)	1.16 *** (1.09–1.24)	0.99 (0.92–1.07)	1.07 ** (1.01–1.14)	0.97 (0.91–1.04)
Legumes and nuts						
Yes	^®^	^®^	^®^	^®^	^®^	^®^
No	1.05 * (0.99–1.12)	1.01 (0.95–1.09)	1.34 *** (1.23–1.46)	1.12 ** (1.01–1.23)	1.27 *** (1.18–1.38)	1.07 (0.98–1.17)
Dairy products						
Yes	^®^	^®^	^®^	^®^	^®^	^®^
No	1.21 *** (1.12–1.3)	1.16 *** (1.07–1.26)	1.38 *** (1.25–1.53)	1.15 ** (1.03–1.29)	1.48 *** (1.34–1.63)	1.24 *** (1.12–1.38)
Flesh foods						
Yes	^®^	^®^	^®^	^®^	^®^	^®^
No	1.15 *** (1.07–1.23)	1.08 * (1–1.18)	1.44 *** (1.3–1.59)	1.17 ** (1.03–1.32)	1.47 *** (1.34–1.61)	1.18 *** (1.06–1.31)
Eggs						
Yes	^®^	^®^	^®^	^®^	^®^	^®^
No	1.18 *** (1.11–1.25)	1.19 *** (1.10–1.28)	1.36 *** (1.25–1.48)	1.11 * (1–1.23)	1.49 *** (1.38–1.62)	1.3 *** (1.18–1.43)
Vitamin A-rich fruits and vegetables						
Yes	^®^	^®^	^®^	^®^	^®^	^®^
No	1.06 ** (1–1.11)	1.05 (0.99–1.12)	1.15 *** (1.07–1.24)	0.93 * (0.85–1.01)	1.12 *** (1.05–1.19)	0.92 ** (0.85–0.99)
Other fruits and vegetables						
Yes	^®^	^®^	^®^	^®^	^®^	^®^
No	1.07 ** (1.02–1.12)	1.1 *** (1.04–1.17)	1.25 *** (1.18–1.34)	1.08 * (0.99–1.16)	1.29 *** (1.22–1.38)	1.20 *** (1.12–1.29)

*** *p* < 0.01, ** *p* < 0.05, * *p* < 0.1; ^a^ Adjusted for age of children, sex of child, currently breastfeeding, diarrhea and fever in last 2 weeks; CI = Confidence Interval in parentheses, OR = Odds Ratio, AOR = Adjusted Odds Ratio, ^®^ = Reference category. Note: HAZ = Height-for-Age Z-scores, WHZ = Weight-for-Height Z-scores, WAZ = Weight-for-Age Z-scores.

## Data Availability

The general datasets are available from the Demographic Health Surveys (DHS) repository. Specifically, the data used for this study are available from the corresponding author upon reasonable request.
